# Macroscopic Characterization of Hepatocellular Carcinoma: An Underexploited Source of Prognostic Factors

**DOI:** 10.2147/JHC.S447848

**Published:** 2024-04-06

**Authors:** Stéphanie Gonvers, Sebastiao N Martins-Filho, André Hirayama, Julien Calderaro, Rebecca Phillips, Emilie Uldry, Nicolas Demartines, Emmanuel Melloul, Young Nyun Park, Valérie Paradis, Swan N Thung, Venancio Alves, Christine Sempoux, Ismail Labgaa

**Affiliations:** 1 Department of Visceral Surgery, Lausanne University Hospital (CHUV), Lausanne, Switzerland; 2 Faculty of Biology & Medicine (FBM), University of Lausanne (UNIL), Lausanne, Switzerland; 3 Department of Laboratory Medicine and Pathology, University of Alberta, Edmonton, Alberta, Canada; 4 Department of Pathology, Faculdade de Medicina FMUSP, Universidade de Sao Paulo, Sao Paulo, Brazil; 5 Department of Pathology, APHP, Henri Mondor University Hospital, Creteil, Val-de-Marne, France; 6 Department of Pathology, Severance Hospital, Yonsei University College of Medicine, Seoul, Republic of Korea; 7 Department of Pathology, APHP, Beaujon University Hospital, Clichy, France; 8 Department of Pathology, Molecular and Cell Based Medicine, Icahn School of Medicine at Mount Sinai, New York, NY, USA; 9 Department of Pathology, Lausanne University Hospital (CHUV), Lausanne, Switzerland

**Keywords:** liver cancer, HCC, prognostication, gross, survival, recurrence

## Abstract

The macroscopic appearance of a tumor such as hepatocellular carcinoma (HCC) may be defined as its phenotype which is *de facto* dictated by its genotype. Therefore, macroscopic characteristics of HCC are unlikely random but rather reflect genomic traits of cancer, presumably acting as a valuable source of information that can be retrieved and exploited to infer prognosis. This review aims to provide a comprehensive overview of the available data on the prognostic value of macroscopic characterization in HCC. A total of 57 studies meeting eligible criteria were identified, including patients undergoing liver resection (LR; 47 studies, 83%) or liver transplant (LT; 9 studies, 16%). The following macroscopic variables were investigated: tumor size (n = 42 studies), number of nodules (n = 28), vascular invasion (n = 24), bile duct invasion (n = 6), growth pattern (n = 15), resection margin (n = 11), tumor location (n = 6), capsule (n = 2) and satellite (n = 1). Although the selected studies provided insightful data with notable prognostic performances, a lack of standardization and substantial gaps were noted in the report and the analysis of gross findings. This topic remains incompletely covered. While the available studies underscored the value of macroscopic variables in HCC prognostication, important lacks were also observed. Macroscopic characterization of HCC is likely an underexploited source of prognostic factors that must be actively explored by future multidisciplinary research.

## Graphical Abstract

**Figure:**
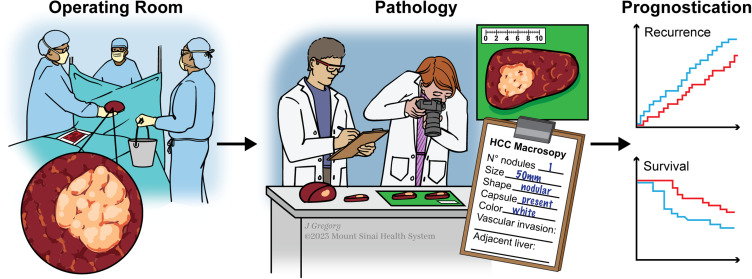

## Introduction

Hepatocellular carcinoma (HCC), the main form of primary liver cancer, is a biologically aggressive malignancy that became a major health problem due to its alarming epidemiological course.^1–3^ Prognostication of HCC has been a particular hurdle. Many research efforts tackled this challenge using various strategies, but the results showed mitigated performances. Naturally, these attempts followed the evolution of other scientific fields and technological breakthroughs such as next-generation sequencing, liquid biopsy or artificial intelligence.^4–6^ One may, however, reasonably wonder whether the contribution of basic input like histology or gross have not been overlooked in this process.

Gross examination is among the first and most simple opportunities to describe and retrieve information from a tumor, in order to infer its prognosis.^7^ The paucity of data on the prognostic value of gross examination in HCC is striking, either because researchers assumed that it was already extensively explored or because they speculated that it was poorly contributive. Although trivial, it is coherent to leverage the genotype-to-phenotype sequence to support that the macroscopic appearance of HCC is unlikely random but rather reflects genomic traits of these tumors and may thus be highly contributive.^8^ Gross examination may be an underestimated and underexploited source of prognostic markers in HCC.

This review aims to provide a comprehensive summary of the available data on the prognostic value of macroscopic characteristics in HCC.

## Materials and Methods

### Search Strategy

An extensive review of the literature was performed to identify articles investigating the prognostic value of macroscopic characteristics in HCC, deriving from gross examination of pathological specimen. Search was conducted in PubMed from inception until 01.01.2023 using the following algorithm: “macroscop*” AND “hepatocellular carcinoma” AND “prognos*”, “gross” AND “hepatocellular carcinoma” AND “prognosis”, “autopsy” AND “hepatocellular carcinoma” AND “prognosis” and “growth pattern” AND “hepatocellular carcinoma”. Cross-referencing was also performed to detect eventual studies not identified by the initial search.

### Eligibility Criteria

Criteria to select eligible studies are detailed in Box 1. The following variables were extracted from selected studies: type of treatment, number of patients and main findings.

**Box 1:** Eligibility Criteria

Human data (in vitro and in vivo studies were excluded). Original data (reviews, commentaries, editorials, systematic review and meta-analyses were excluded). Studies reporting macroscopic characteristics retrieved from pathological analysis of specimen (surgical resection, transplantation or autopsy). Studies retrieving macroscopic variables only based on imaging were excluded. Analysis of the prognostic value of macroscopic variables on either survival or recurrence. The most recent and complete article was chosen if a study had been published more than once. Available full-text publications in English.

### Data Analysis

Forest plots were generated to illustrate associations between macroscopic HCC characteristics and prognosis (ie, recurrence and overall survival (OS)).

## Main Findings

**Figure 1:**
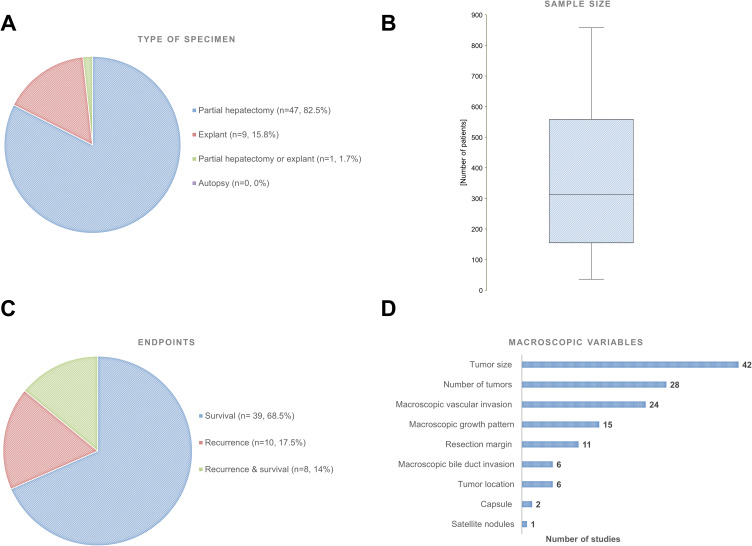
Characteristics of the eligible studies. (**A**) Types of specimen grossly examined. (**B**) Boxplot representing the distribution of sample size. (**C**) Endpoints investigated. (**D**) Types of macroscopic variables analyzed.

A total of 57 studies met eligibility criteria. Macroscopic characterization of HCC derived from gross analysis of partial hepatectomy (47 studies, 82.5%) or explants (9 studies, 15.8%), whereas 1 study (1.7%) included patients undergoing either liver resection (LR) or liver transplant (LT). Of note, no study using autopsy was identified (Figure 1A). The median sample size was 313 patients [155–559] (Figure 1B). Recurrence and overall survival (OS) were selected as endpoints to investigate potential association between HCC macroscopy and prognosis in 10 (17.5%) and 39 (68.5%) studies, respectively (Figure 1C). Investigated macroscopic variables included tumor size (n = 42 studies), number of nodules (n = 28), vascular invasion (n = 24), bile duct invasion (n = 6), growth pattern (n = 15), resection margin (n = 11), tumor location (n = 6), capsule (n = 2) and satellite (n = 1) (Figure 1D). Summary of the findingswas provided for each of these macroscopic features, hereunder.

### Tumor Size

Tumor size (TS) is a widely explored parameter in HCC, integrated to a certain extent – in several staging systems and classifications such as the American Joint Committee for Cancer (AJCC), Tumor-Node-Metastasis (TNM),^9^ Milan criteria,^10^ or Barcelona Clinic Liver Classification (BCLC).^11^

Imaging modalities have shown high sensitivity to measure the size of HCC nodules and good correlation with gross examination.^12^ Therefore, size of HCC reported in the literature is commonly based on imaging. Herein, we focused on studies reporting size based on gross examination.

TS was the most extensively investigated macroscopic item (Figure 1D), assessed in 42 available studies, detailed in Table S1.

#### Recurrence

A total of 15 articles focused on the association with recurrence.

A recent study including 698 HCC patients undergoing LR showed that TS >10 cm was an independent prognostic factor of early recurrence within 2 years with an odds ratio (OR) of 1.72 (95% CI, 1.20–2.46; p = 0.003).^13^ After inclusion of size in nomograms, the predictive accuracy was confirmed in both training and validation cohorts. Of note, it included only patients without macroscopic vascular invasion (MaVI). In a large-sample study including 734 patients after LR, TS above 5 cm was also identified as a predictor of late recurrence (ie, >2 years after surgery) with an increased hazard ratio (HR) of 1.49 (95% CI, 1.10–2.00; p = 0.009).^14^ Likewise, as a continuous variable in a Western multicentric study including 441 patients, TS on explants was associated with recurrence (HR, 1.73; p < 0.05).^15^

**Figure 2:**
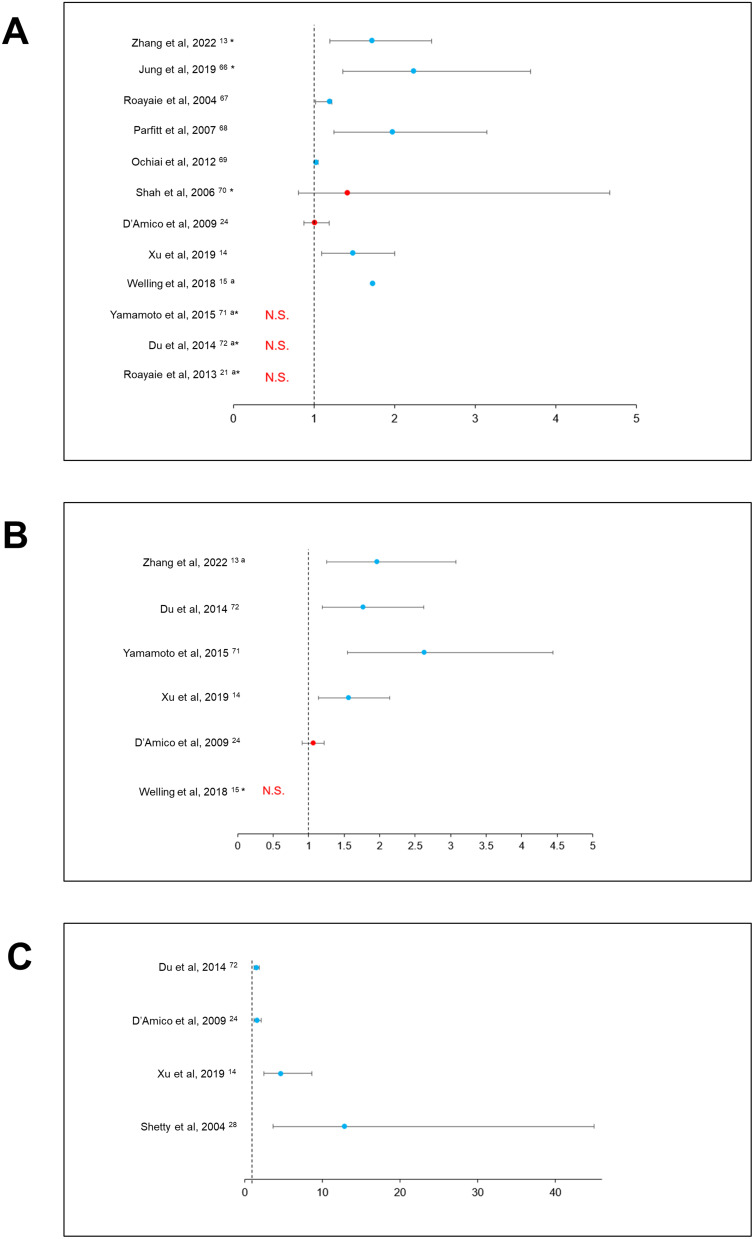
Associations between macroscopic features and recurrence. (**A**) Forest plot illustrating the association of tumor size with recurrence. *Size was analyzed as a dichotomized variable ªDetailed results not provided. (**B**) Forest plot illustrating the association of number of nodules with recurrence. ªComparison of 2 vs 1 nodule *Detailed results not provided. (**C**) Forest plot illustrating the association of macroscopic vascular invasion with recurrence.

**Abbreviations**: N.S., not significant.

Among articles providing multivariable analyses, TS was associated with recurrence in 7/12 (58%) studies (Figure 2A).

#### Survival

The association between TS and OS was analyzed in 23 studies.

A large-scale Japanese study including 13’566 resected patients reported a significant association of size >2 cm and worse survival (RR, 1.21; 95% CI, 1.14–1.28; p < 0.0001).^16^ The dichotomization of size with a cut-off of 5 cm yielded similar findings, in other studies.^17–20^ For this reason, Poon et al suggested a shift in the cut-offs used in classifications, from 2 to 5 cm.^19^ Using dichotomization with cut-offs varying from 3 to 7 cm, other studies showed comparable results for OS.^21–23^

**Figure 3:**
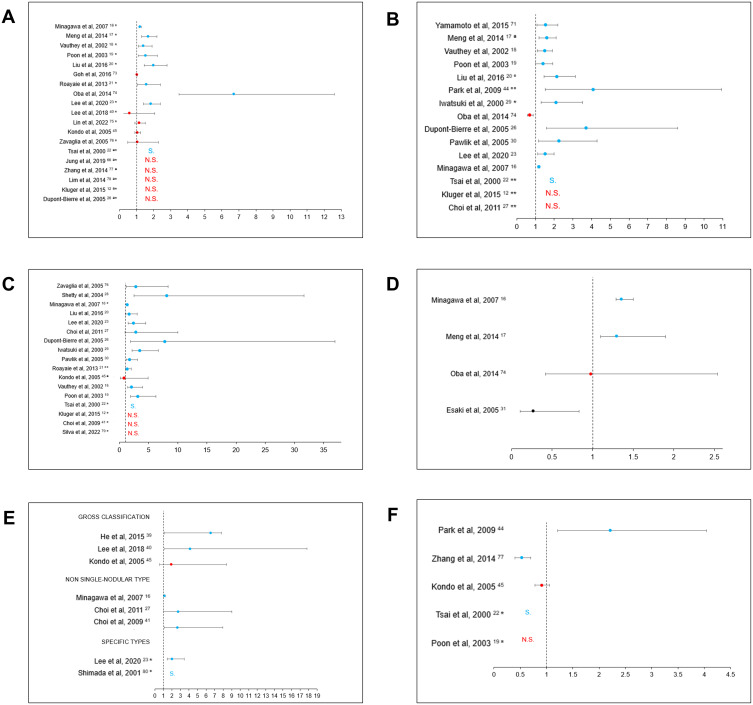
Associations between macroscopic features and overall survival (OS). (**A**) Forest plot illustrating the association of tumor size with OS. *Size was analyzed as a dichotomized variable. ªDetailed results not provided. (**B**) Forest plot illustrating the association of number of nodules with OS. ªNoN ≥ 2°NoN ≤ 3 °°NoN > 3 *NoN 1–2 vs 3–4. **Detailed results not provided. (**C**) Forest plot illustrating the association of macroscopic vascular invasion with OS. °Vascular or bile duct invasion.°°Extent to hepatic vein or inferior vena cava. ªInvasion along portal vein system.*Detailed results not provided. (**D**) Forest plot illustrating the association of macroscopic bile duct invasion with OS. (**E**) Forest plot illustrating the association of macroscopic growth pattern with OS. *Association between infiltrative type and OS. °Association between single-nodular with extra-nodular growth and OS (detailed results not provided). (**F**) Forest plot illustrating the association of resection margin with OS. *Detailed results not provided.

**Abbreviations**: S, Significant; N.S., not significant.

The available multivariable analyses identified TS as an independent prognostic factor of OS in 9/19 studies (47%) (Figure 3A).

### Number of Nodules

Number of nodules (NoN) or so-called focality is also a well-established parameter to assess tumor burden. Our review compiled 28 studies analyzing its prognostic impact, as listed in Table S2.

#### Recurrence

The risk of recurrence according to NoN was analyzed by 9 studies.

In a Chinese multicentric study with 734 patients, NoN was associated with an increased risk of relapse after LR (HR, 1.56; 95% CI, 1.14–2.14; p = 0.006).^14^ Conversely, in a Western multicentric study including 479 HCC transplanted patients mentioned previously, NoN found in explants had no association with recurrence (p = 0.40).^24^

A higher risk of recurrence due to multifocality was highlighted in 4/6 (67%) studies (Figure 2B).

#### Survival

A total of 17 studies exploring the impact of NoN on OS were selected.

Its prognostic value, as a continuous variable, was detected in a large-scale Japanese cohort including 13’566 patients after LR, showing an increased RR of 1.18 (95% CI, 1.12–1.23; p < 0.0001).^16^ In a study with 300 HCCs >10 cm, analysis was performed within a statistical model integrating macrovascular invasion, degree of fibrosis and AFP level, as potential confounding factors. Even in this context, a significant association between NoN and OS was proven with an HR of 2.25 (95% CI, 1–17-4.30; p = 0.02).^25^

Overall, 12/15 (80%) studies investigating OS in the context of multifocal HCC reported an association (Figure 3B).

### Macroscopic Vascular Invasion

MaVI, commonly defined as portal and/or hepatic vein invasion, is an important parameter that, when identified radiologically or intra-operatively, typically contraindicates surgical treatments.

The performance of imaging to detect MaVI has gradually improved and is now standard. MaVI is integrated into grading score systems as it could help stratifying patients and guide treatment-strategy.^24^

Our review includes 24 studies assessing the prognostic value of MaVI (Table S3).

#### Recurrence

The potential implication of MaVI in HCC recurrence was studied in 5 articles.

Analysis of 479 explants identified microvascular invasion, histological grade and MaVI (HR, 1.58; 95% CI, 1.17–2.10; p = 0.03) as independent predictors of recurrence after LT.^24^ After LR on 734 patients, MaVI also appeared to increase the risk of late recurrence (ie, >2 years of surgery) with an HR of 4.63 (95% CI, 2.48–8.67; p < 0.001).^14^

MaVI, regardless of its precise location, appeared to dictate recurrence after any type of surgical treatment (4/4 studies, 100%) (Figure 2C).

#### Survival

Eighteen studies investigated the association between MaVI and OS.

As expected, the deleterious prognostic impact of MaVI was demonstrated by numerous studies.^16^,^20^,^22^,^23^,^26–30^ As an example, Roayaie et al investigated the outcomes of a cohort of 165 patients, with macrovascular invasion undergoing LR.^21^ Extent of tumor to hepatic veins or inferior vena cava was identified as a prognostic factor of poorer OS (HR, 1.32; 95% CI, 1.03–1.74; p = 0.05). This subgroup of patients had a median survival of only 4.7 months compared to 9.2 months for patients with MaVI to main portal vein and 15.5 months for patients without any MaVI (p < 0.001). Furthermore, they showed a surprisingly high perioperative rate of 28%, compared to 6% for HCC expanding to main portal vein and 3% for tumor limited to segmental of lobar branches (p < 0.001).

However, the same study found that invasion in the main portal vein was not associated with survival (data not available).

Altogether, regardless of the site of invasion, 13 out of 17 studies (76%) found an association of presence of macroscopic vessel invasion with poor OS (Figure 3C).

### Macroscopic Bile Duct Invasion

Due to their anatomical relation and proximity, invasion of portal branches and bile ducts commonly co-occur.

Six studies focused on macroscopic bile duct invasion (MaBDI) and its impact on OS (Table S4).

Meng et al conducted a study on 413 resected patients, aiming to explore the prognosis of BDI. A central type of BDI, defined as the invasion of common hepatic duct or first-order branch of bile ducts with or without microscopic invasion of intrahepatic peripheral bile duct, was proven to be an independent prognostic factor of poorer OS (HR, 1.3; 95% CI, 1.1–2.2; p = 0.01) rather than a peripheric invasion.^17^

In a cohort including only 38 patients undergoing LR, Esaki et al reported a protective effect of MaBDI, compared to patients with microscopic bile duct invasion (HR, 0.27; 95% CI, 0.11–0.67; p = 0.005).^31^ This could be explained by the fact that patients with MaBDI underwent a wider extent of liver resections, compared to the ones with only microscopic bile duct invasion.

Overall, the association between MaBDI and poor survival was highlighted by 2/4 (50%) (Figure 3D).

### Growth Pattern

Okuda et al first described HCC gross pathologic patterns using autopsy material from Japan, United States and South Africa.^32^ Of note, no prognostic analysis was performed. They classified tumor in three subtypes: expanding, spreading and multifocal. In addition, incidence discrepancies between the three types were noticed between geographic areas. There are very few data on the prognostic significance of growth pattern (GP).^33^ To date, there are only two gross classifications of HCC: one elaborated by the Liver Cancer Study Group of Japan (LCSGJ) and the other by the Korean Liver Cancer Association (KLCA). The LCSGJ classification divided tumors in four categories: (I) single nodular, (II) single nodular with extra-nodular growth, (III) confluent multinodular, and (IV) infiltrative.^34–36^ Likewise, the KLCA simplified HCC classification into five categories: (I) vaguely nodular, (II) expanding nodular, (III) multinodular confluent, (IV) nodular with peri-nodular extension and (V) infiltrative.^8^,^36^

Fifteen studies analyzed the prognostic value of these gross classifications, of which only 12 performed multivariate analyses (Table S5). Of note, all of them were conducted in Eastern cohorts.

#### Recurrence

Only 2 studies investigated the association with recurrence.^37^,^38^ In a Korean study with 266 resected HCCs, an increased risk of tumor relapse was showed for the confluent multinodular (HR 2.61; 95% CI, 1.26–5.41; p = 0.010) and infiltrative types (HR 3.62; 95% CI, 1.30–10.05; p = 0.014).^38^

#### Survival

A total of 10 studies explored the impact of GP on OS.

Overall, gross classification was an independent prognostic factor of poorer OS (RR, 6.56; 95% CI, 1.12–2.37; p = 0.01).^39^ Several articles chose to divide gross types into single nodular and non-single nodular types.^16^,^27^,^40^,^41^ This way, in a previously mentioned study, Minagawa et al showed that non-single nodular types were independently predictive of lower OS with an increased RR of 1.13 (95% CI, 1.08–1.18; p < 0.0001).^16^

Some studies focused on each gross type separately. In a cohort including 144 patients with solitary HCCs, infiltrative type was associated with the worst 5-year survival rate (51.5% vs 70–80% for the other types, p < 0.05).^39^

In a cohort of 242 patients undergoing LR, Lee et al demonstrated a deleterious impact of three subtypes (confluent multinodular, nodular with peri-nodular extension and infiltrative) on survival with an HR of 4.12 (95% CI, 1.14–14.84; p = 0.03).^40^ Moreover, these subtypes were associated with clinicopathological features of aggressive biological behavior such as poor histological differentiation (p = 0.001) or more frequent microvascular invasion (p < 0.001). Additionally, the same subtypes correlated with an increased expression of “stemness-” and EMT-related markers, such as EpCAM (p = 0.009), CK19 (p = 0.002), uPAR (p<0.001), and ezrin (p = 0.036) which could explain highly invasive potential.

Overall, GP was found as a predictive factor of survival in 7/8 (87.5%) studies (Figure 3E).

Of interest, the ability to predict gross classification based on preoperative imaging was assessed in 2 studies.^37^,^42^ Using CT-scan, He et al reported an accuracy of 65.3%, with 5 mm-thickness slices,^39^ whereas a correlation coefficient of 86.9% was obtained with ultrasound.^42^ Of note, these independent and different studies do not allow comparison between both techniques.

### Resection Margin

Eleven studies focused on this variable (Table S6). As underpinned by Chau et al, measurement should be performed before formalin fixing.^43^

#### Recurrence

Three studies analyzed impact on recurrence but only one performed multivariable analysis. This multicentric study included 734 patients who underwent LR.^14^ After dichotomization, a resection margin (R) of less or equal to 1 cm was not associated with late recurrence (i.e., >2 years after surgery) (p = 0.25).

#### Survival

A total of 9 studies focused on the association between R and OS, of which 5 performed multivariate analyses.

Four studies analyzed a margin of less versus more than 1 cm. In a study including 213 Child-Pugh A patients undergoing LR, Park et al showed an increased OR of 2.21 (95% CI, 1.21–4.05; p = 0.01) for the group with R >1cm.^44^

Conversely, as a continuous variable in a cohort of 110 resected patients, R was not a prognostic factor of survival (HR, 0.91; 0.78–1.06; p = 0.23).^45^ Of note, this study excluded the confluent multinodular and infiltrative types.

Altogether, the role of surgical margin in predicting patient’s survival still remains controversial, since the few available studies have been based on non-standardized criteria and only 3/5 (60%) found a deleterious association (Figure 3F).

### Tumor Location

Few studies investigated whether the location of HCC nodules may be associated with prognosis. We found 4 studies comparing unilobular HCC to bilobar HCC (Table S7).

Recently, a group of researchers from China studied whether the anatomic location of HCC affected survival after LR in a cohort of 700 patients.^46^ They observed that left liver tumors were a prognostic factor of long-term worse OS (HR, 3.23; 95% CI, 1.28–8.13; p = 0.013).^46^

### Capsule Formation

Tumor capsule formation (CF) was assessed in only 2 studies (Table S8). One of them conducted a multivariate analysis on 322 resected HCCs but did not find any association between macroscopic encapsulation and OS (p = 0.40).^22^ In analogy with neoplasms in other solid organs, a clear definition of encapsulated patterns of HCC is expected to yield relevant further information

### Satellite Nodules

Satellite nodules are defined as smaller HCC nodules separated from the primary tumor by non-cancerous tissue with a distance ≤2 cm.^47^ Satellites are widely explored in the literature. However, it is rarely specified whether they were detected on gross or on histology.^14^,^26^,^41^,^46^

A single study specifically mentioned it assessed the presence of satellite lesions on gross pathological findings and defined them as “small lesions located in the vicinity of the resected tumor and undetected by imaging” (Table S9).^12^ It included 313 patients of which 134 had macro satellites nodules. Overall, these daughter nodules were independently predictive factor of poorer OS (HR, 1.69; 95% CI, 1.14–2.50; p = 0.009).

## Discussion

This review summarized the available data on the prognostic role of macroscopy in HCC.

Results unveiled two main findings: data on the topic remain sparse and there are numerous gaps deserving to be further explored and filled. Regarding the former, only 57 studies were found, a lack that becomes particularly striking when considering that gross examination of HCC has been performed for many decades.^7^,^8^ For the latter, the interest and the quality of the selected studies did not prevent several important drawbacks. Shortcomings have been identified at different levels: in reporting gross examination and within the analytical phase. Details on macroscopic variables were commonly missing. As examples, studies rarely detailed whether data (ie, TS, NoN, MaVI and other variables) derived from gross examination or from preoperative imaging. Likewise, it is frequently unclear and unspecified whether tumor characteristics such as MaBDI or satellites were based on histology or gross examination. As macroscopic items presumably have a pivotal significance for patients’ outcomes, it would be valuable to assess them more thoroughly; as an example, the distinction between portal and hepatic vein invasions in MaVI may be insightful.^21^ Finally, most studies were conducted in Asian cohorts. It is thus difficult to know whether they may be extrapolated to Western HCC.^48^

Results on GP revealed unexpected gaps, identifying few studies and only two classifications established in cohorts of Eastern HCC.^36^ Both classifications were quite categorical and only proposed 4–5 subclasses. A thorough and systematic assessment of gross HCC – including a precise list of criteria – may offer increased reproducibility and granularity.^8^

Several limitations of the analytical approaches were also noted. First, most studies lacked validation sets. This is an important point considering the high risk of confounding factors. The numerous interactions between the different variables are difficult to deconvolute and there is a strong risk of collinearity.^24^ R perfectly illustrates the lack of standardization in reporting and analyzing gross findings: it has been analyzed either as a continuous or as a categorical variable. When dichotomized, various thresholds were applied. The concept of “narrow margin” defined as R ≤ 1 cm has been investigated on multivariable analysis in only 1 and 4 studies for recurrence and OS, respectively.

Finally, statistical analyses were basic. Although it is not per se a flaw, it would be interested to conduct more sophisticated analyses such as scores, in order to combine the prognostic input of different variables, and to therewith optimize performances. Such scores should be thereafter tested in multivariable models including variables such as the Child-Pugh score and other biomarkers or scores.^49–51^

In term of perspectives, there is a critical need to improve and standardize gross reporting.^47^ Graphical Abstract illustrates the typical workflow between the operating theater and the core of pathology. Although efforts were made from entities such as CAP and ICCR,^47^,^52^ further guidelines determining which and how macroscopic variables should be reported are yet required. These datasets would offer precious information that can be subsequently submitted to conventional or artificial intelligence-based analyses, for prognostication. Also, it would be of paramount value to leverage the genotype-to-phenotype paradigm and thoroughly investigate the potential link between molecular and macroscopic features of HCC. Recent studies were able to identify molecular subclasses of HCC with specific macroscopic traits.^53–57^ A recent insightful study including 400 HCC established a classification with 4 subtypes of HCC, based on macroscopy. Of note, the classification derived from the Japanese one – previously described^34^ – but also included margin status. Using multiomics analysis, authors showed that the gross subtypes displayed distinct transcriptional patterns.^48^

Of importance, macroscopic characterization may not only show valuable input for prognostication, it may also have important contribution for treatment allocation, a major point in terms of clinical significance. Recent data on the application of artificial intelligence on histology have shown very promising results, and the same concept may also be applied to macroscopy.^58–60^ Of course, macroscopy is only available after surgical treatments and is unfortunately unavailable for patients treated with other therapeutical options for which important progress has been made recently.^61–63^ If future studies confirm the prognostic value of macroscopy, it will be needed to thereafter determine how it can be integrated into guidelines, such as the Barcelona Clinic Liver Cancer (BCLC) classification.^64^ As an example, gross findings may offer opportunities to select patients who will benefit from adjuvant treatments after LR or LT, as these therapeutical strategies will hopefully become available.^65^

The present study is not strictly a systematic review. Although it was conducted according to a specific and detailed methodology, not all databases were queried. Thus, available studies may have potentially been missed.^66^

## Conclusion

In summary, this unprecedented review on the prognostic contribution of macroscopic characterization of HCC showed that data on the topic are scarce. Available studies showed interesting results that revealed the value of macroscopic features to prognosticate HCC. However, it also highlighted some limitations and above all some important gaps deserving to be addressed by future research efforts. These findings support the idea that macroscopy is an underexploited source of information in HCC patients. The results also stress the need to attach a particular importance to macroscopy and to intensify research on the topic.

## Abbreviations

HCC, hepatocellular carcinoma; OS, overall survival; LR, liver resection; LT, liver transplant; TS, tumor size; AJCC, American Joint Committee for Cancer; TNM, Tumor-Node-Metastasis; BCLC, Barcelona Clinic Liver Classification; MaVI, macroscopic vascular invasion; NoN, number of nodules; MaBDI, macroscopic bile duct invasion; GP, growth pattern; LCSGJ, Liver Cancer Study Group of Japan; KLCA, Korean Liver Cancer Association; R, resection margin; CF, capsule formation.
